# Supplementary material for: The interactome of intact mitochondria by cross-linking mass spectrometry provides evidence for coexisting respiratory supercomplexes
Source: Mol Cell Proteomics. 2017 Dec 8;17(2):216–32. doi: 10.1074/mcp.RA117.000470 (PMC5795388; doi:10.1074/mcp.RA117.000470)
Supplement: Supplemental Data [file supp_RA117.000470_133922_0_supp_23978_fzffwf.zip › spectra_annotation/mito_spectra_annotation/407-1-9-1-6-1.pdf]

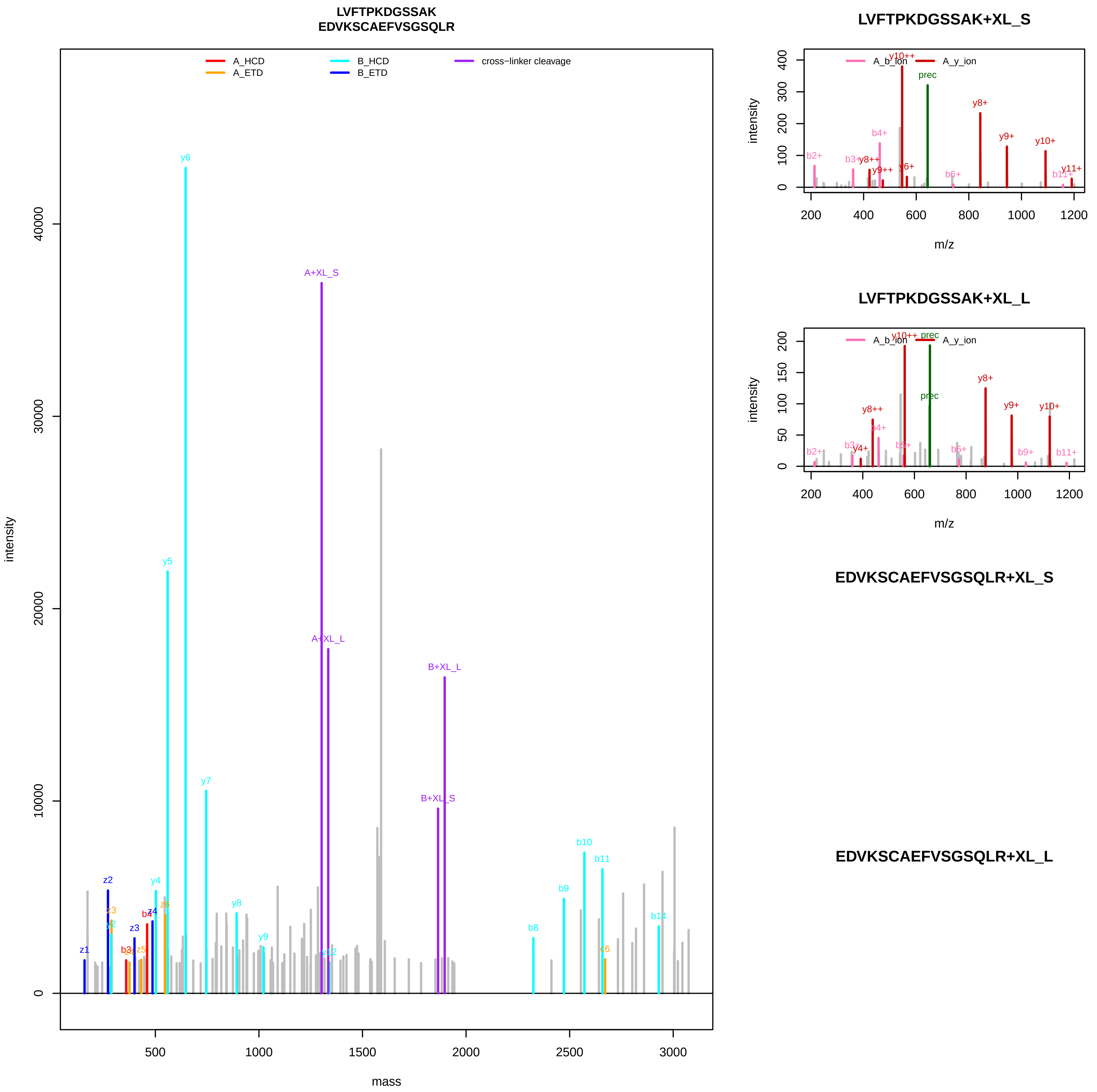
LVFTPKDGSSAK
EDVKSCAEFVSGSQLR
A_HCD
A_ETD
B_HCD
B_ETD
cross−linker cleavage
y6
A+XL_S
A+XL_L
B+XL_L
B+XL_S
y5
y7
b10
b11
b9
y4
z2
z3
z4
b4
z5
z6
y8
y9
y2
z1
b3
b8
b14
c6
y12
intensity
mass
0
10000
20000
30000
40000
500
1000
1500
2000
2500
3000
LVFTPKDGSSAK+XL_S
A_b_ion
A_y_ion
prec
y10++
y8+
y9+
y10+
y11+
b2+
b3+
b4+
b6+
b11+
y8++
y9++
y6+
intensity
m/z
LVFTPKDGSSAK+XL_L
y10++
prec
y8+
y9+
y10+
y8++
y4+
b2+
b3+
b4+
b5+
b6+
b9+
b11+
intensity
m/z
200
400
600
800
1000
1200
EDVKSCAEFVSGSQLR+XL_S
EDVKSCAEFVSGSQLR+XL_L
